# A novel predictive model of microvascular invasion in hepatocellular carcinoma based on differential protein expression

**DOI:** 10.1186/s12876-023-02729-z

**Published:** 2023-03-27

**Authors:** Zhenglu Wang, Lei Cao, Jianxi Wang, Hanlin Wang, Tingting Ma, Zhiqi Yin, Wenjuan Cai, Lei Liu, Tao Liu, Hengde Ma, Yamin Zhang, Zhongyang Shen, Hong Zheng

**Affiliations:** 1 Biological Sample Resource Sharing Center, Tianjin First Central Hospital, Nankai University, Tianjin, China; 2 Department of Pathology and Laboratory Medicine, University of California in Los Angeles (UCLA), Los Angeles, CA USA; 3 Pathology Department, Tianjin First Central Hospital, Nankai University, Tianjin, China; 4 Research Institute of Transplant Medicine, Nankai University, Tianjin, China; 5 Key Laboratory of Transplant Medicine, Chinese Academy of Medical Sciences, 24 Fukang Road, Nankai, Tianjin, 300192 China; 6 HPS Gene Technology Co., Ltd., Tianjin, China; 7 Organ Transplant Department, Tianjin First Central Hospital, Nankai University, Tianjin, China; 8 Tianjin Key Laboratory for Organ Transplantation, Tianjin First Central Hospital, Nankai University, Tianjin, China

**Keywords:** Hepatocellular carcinoma, Microvascular invasion, Differential protein expression, Risk factors, Predictive model

## Abstract

**Background:**

This study aims to construct and verify a nomogram model for microvascular invasion (MVI) based on hepatocellular carcinoma (HCC) tumor characteristics and differential protein expressions, and explore the clinical application value of the prediction model.

**Methods:**

The clinicopathological data of 200 HCC patients were collected and randomly divided into training set and validation set according to the ratio of 7:3. The correlation between MVI occurrence and primary disease, age, gender, tumor size, tumor stage, and immunohistochemical characteristics of 13 proteins, including GPC3, CK19 and vimentin, were statistically analyzed. Univariate and multivariate analyzes identified risk factors and independent risk factors, respectively. A nomogram model that can be used to predict the presence of MVI was subsequently constructed. Then, receiver operating characteristic (ROC) curve, calibration curve, and decision curve analysis (DCA) were conducted to assess the performance of the model.

**Results:**

Multivariate logistic regression analysis indicated that tumor size, GPC3, P53, RRM1, BRCA1, and ARG were independent risk factors for MVI. A nomogram was constructed based on the above six predictors. ROC curve, calibration, and DCA analysis demonstrated the good performance and the clinical application potential of the nomogram model.

**Conclusions:**

The predictive model constructed based on the clinical characteristics of HCC tumors and differential protein expression patterns could be helpful to improve the accuracy of MVI diagnosis in HCC patients.

**Supplementary Information:**

The online version contains supplementary material available at 10.1186/s12876-023-02729-z.

## Background

Globally, liver cancer is the sixth most common cancer and the fourth leading cause of cancer death, of which hepatocellular carcinoma (HCC) accounts for about 75%–85% [1]. Although the treatment of HCC has witnessed continuous improvements in the past few decades, the 5-year recurrence rate is still > 70%, making the overall prognosis of liver cancer patients unsatisfactory [2]. Microvascular invasion (MVI) is an important factor affecting the recurrence of HCC, and can be used as an important predictor of the prognosis of HCC patients [3, 4]. The accurate determination of MVI could help clinically determine the tumor stage, make appropriate treatment plans and improve the prognosis of the patients. Pathological examination is the “gold criteria” of MVI diagnosis, but it suffers a tendency to under-report the incidence of MVI in HCC as the limited scope of routine glass slides and randomness and bias of sampling. Therefore, constructing a reliable prediction model as an auxiliary diagnostic tool will improve the accuracy of MVI diagnosis.

In recent years, domestic and foreign studies have found that serum alpha-fetoprotein (AFP) > 400 μg/L and prothrombin (proteins induced by vitamin K absence of antagonist, PIVKA-II) > 90 mAU/mL were independent risk factors for MVI [5]. Enolase 1 (ENO1) and heat shock protein 70 (HSP70) antibody levels were observed to be predictive biomarkers of MVI [6]. In addition, the up-regulation of microRNA-125b, lncRNA AWPPH, and kindlin-2 protein expression in the serum were closely related to the occurrence of MVI [7]. Although some progress has been made in predicting preoperative MVI in terms of serological indicators and genetic characteristics, its diagnostic accuracy is low and clinical use is inconvenient.

The protein expression can more accurately reflect its biological characteristics in HCC tissue, which could help to improve the accuracy of MVI diagnosis [8]. The prediction of MVI by protein expression changes in tumor tissue may have higher specificity and sensitivity than serological markers. At present, logistic regression analysis has shown great potential in tissue sample analysis, which can effectively improve the objectivity and accuracy of the diagnosis [9]. However, there is no relevant study based on this analysis to predict the MVI based on the clinical characteristics and differential protein expression patterns in HCC patients. Therefore, 200 patients with HCC were selected and 13 protein expression patterns closely related to the prognosis of HCC were detected [10, 11]. Based on the clinical and tumor characteristics of HCC, we applied logistic regression analysis to ascertain the risk factors, constructed a nomogram model to predict MVI, and assessed its clinical application value for evaluating MVI.

## Methods

### Patients

**Fig. 1:**
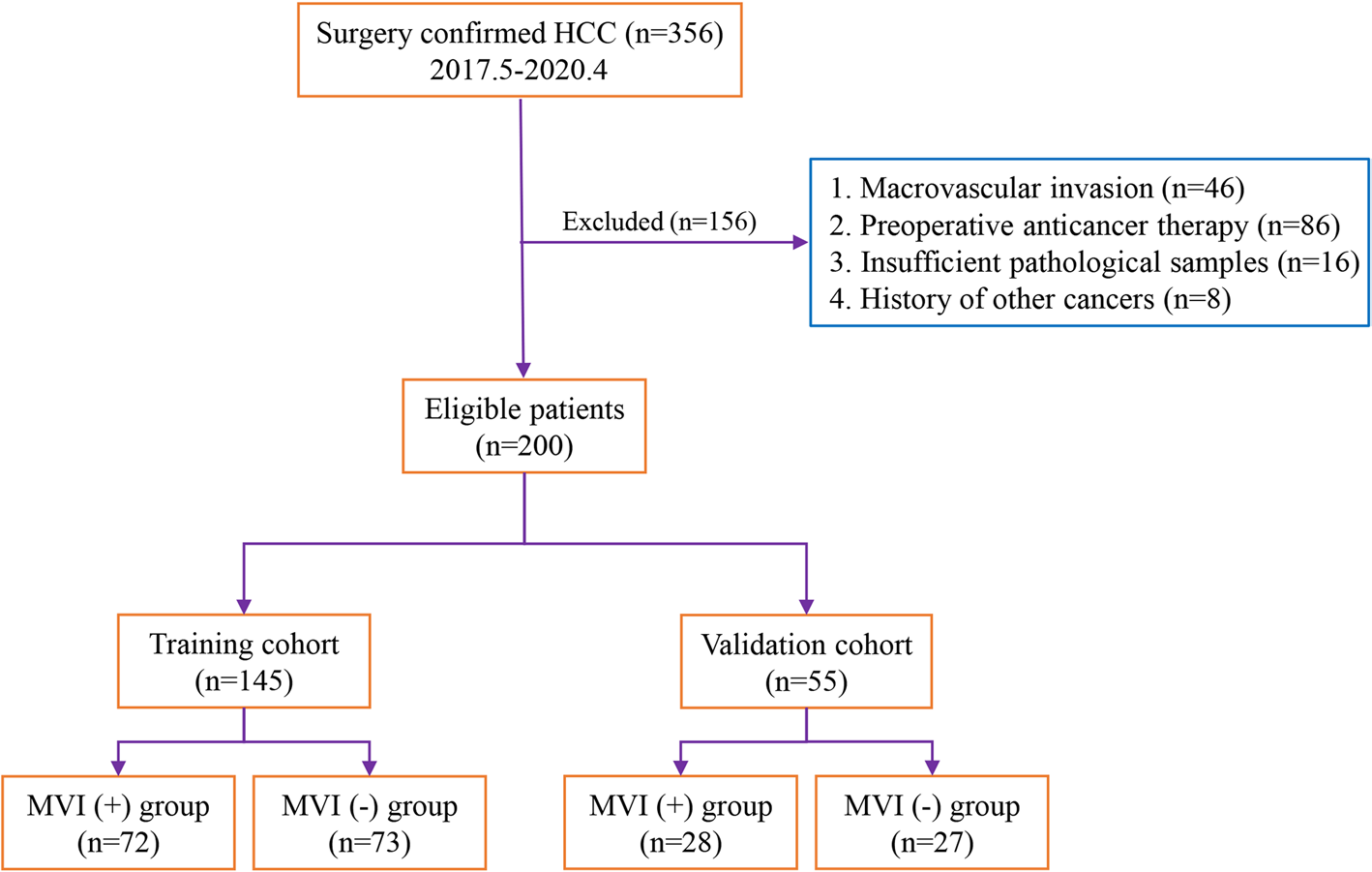
Flow chart of the research population selection

The study was approved by the Ethics Committee of the First Central Hospital Affiliated to Nankai University. All patients signed an informed consent form. Records of patients with HCC who underwent surgical resection from May 2017 to April 2020 were collected. The screening process and the inclusion and exclusion criteria was depicted (Fig. 1). Data pertaining to age, gender, background disease (Fig. S1), tumor size, tumor differentiation, presence of MVI. All samples were randomly divided into training set and validation set according to the ratio of 7:3 in the following study.

### Diagnostic criteria

All cases were confirmed pathologically as HCC, and the tumor histological differentiation was determined. The diagnostic criteria were in accordance with the HCC pathological diagnostic criteria and histological differentiation criteria listed in the 5^th^ edition of WHO 2019 Digestive System Tumors [12]. The MVI diagnostic criteria were as follows: microscopic determination of tumor cell clusters in the blood vessels or lymphatic vessels along with endothelial cells in the tumor capsule and/or tumor margin and/or adjacent tissues. As tumor cell clusters often adhere to or invade the vascular wall, those in the blood sinus in the tumor tissues were not identified as MVI [13].

### Detection of protein expression in tumor tissues

Immunohistochemical staining was used to detect the expression patterns of GPC3, CK19, vimentin, p53, AFP, EGFR, RRM1, BRCA1, VEGF, TS, Ki 67, ARG and GS in HCC tissues. The staining procedure was performed according to the instructions of the Roche benchmark XT Ventana (Roche Diagnostics, USA) automatic staining instrument, and the paraffin sections were detected by automatic immunohistochemistry. The slices were dewaxed, pretreated with cleaning buffer for 30 min, and then washed with reaction buffer. Subsequently, the following primary antibodies were added: GPC3, CK19, vimentin, mutant p53, AFP, EGFR, VEGF (all sourced from Santa Cruz Biotechnology, USA), RRM1, and BRCA1 (both sourced from Abcam, UK), After incubation for 30 min, the cells were stained with ultraView Universal DAB Kit (Roche Diagnostics, GER).

Positive criteria: All immunohistochemical staining results were judged independently by two senior pathologists, and unanimous interpretations are made in the event of a disagreement. The agreement between the two experts on the positive results of immunohistochemical staining was 97.15% (2526/2600). Furthermore, 74 immunohistochemical stained sections with inconsistent judgments were agreed upon after consultation.

### Construction and validation of the nomogram model

(1) In the training cohort, if the univariate difference was statistically significant (*p* < 0.1 was taken as the threshold for inclusion in the multivariate analysis), the factor was included in the multivariate model for analysis. (2) In the multivariate analysis, binary multiple logistic regression was used and forward Wald was selected to screen the independent risk factors for MVI. (3) The rms package of R was employed to construct the nomogram model on the basis of the above risk factors, and the nomogram was drawn to evaluate the relationship between the predictive and actual probabilities. (4) ROC curve was drawn to assess the discrimination of predictive score in the training and validation cohorts. (5) Hosmer–Lemeshow goodness-of-fit test and calibration curve were employed to evaluate the calibration in the two cohorts, with two-sided *p* < 0.05 indicating statistical significance. (6) DCA analysis was utilized to estimate the clinical validity and net benefit of the model.

### Statistical analysis

SPSS 26.0 software and R version 3.6.1 were applied to analyze the data. The measurement data were displayed in the form of mean ± standard deviation, and the comparison between the two groups was carried out using independent samples t-test. The classification data have been presented in the form of numbers and percent (%). The comparison between the two groups was performed using Chi-square test, and Fisher's test was used when the exact probability met.

## Results

### Clinical characteristics

A total of 200 patients were enrolled, including 147 men (73.5%) and 53 women (26.5%) aged 36–65 years (mean age 53.67 ± 9.91). The clinical characteristics of the patients in the training and validation cohorts were displayed (Table S1). MVI (-) accounted for 50.34% and 49.09% in the training and validation cohorts, respectively. When MVI was present, there was no statistically significant difference between the two groups (*p* = 0.972). In addition, there were no obvious differences in the other clinical characteristics between the two groups. This result justified the rationality of using the training and validation cohorts.

### Analysis of risk factors for MVI

Univariate analysis revealed that tumor size (odds ratio [OR] = 19.317, *p* < 0.001), Ts (OR = 3.054, *p* = 0.001), GPC3 (OR = 3.864, *p* < 0.001), P53 (OR = 1.808, *p* = 0.081), RRM1 (OR = 3.331, *p* = 0.001), BRCA1 (OR = 3.612, *p* = 0.001), and ARG (OR = 0.394, *p* = 0.007) were the risk factors for MVI. The difference was statistically significant (*p* < 0.1) (Table 1). The results suggested that tumor size, Ts, GPC3, P53, RRM1, BRCA1, and ARG were closely related to the occurrence of MVI.

**Table 1:** Univariate logistic regression analysis of MVI occurrence in the training cohort

Variable	B	S.E	*P*	OR (95%CI)
Age	-0.018	0.017	0.308	0.983(0.949–1.016)
Gender	-0.267	0.379	0.481	0.765(0.361–1.607)
Tumor size	2.961	0.429	< 0.001	19.317(8.624–46.668)
Tumor differentiation	-18.660	1110.900	0.987	7.887e-09(3.491e-155–6.478e + 14)
GPC3	1.352	0.352	< 0.001	3.864(1.958–7.828)
CK19	0.194	0.333	0.561	1.213(0.632–2.336)
vimentin	-0.304	0.334	0.363	0.738(0.382–1.419)
P53	0.592	0.340	0.081	1.808(0.933–3.543)
AFP	0.248	0.334	0.458	1.282(0.666–2.476)
EGFR	0.260	0.337	0.441	1.296(0.670–2.522)
RRM1	1.203	0.353	< 0.001	3.331(1.685–6.759)
BRCA1	1.284	0.379	< 0.001	3.612(1.748–7.781)
VEGF	0.031	0.334	0.927	1.031(0.535–1.988)
Ts	1.116	0.347	0.001	3.054(1.561–6.106)
Ki 67	-0.486	0.367	0.186	0.615(0.296–1.258)
ARG	-0.932	0.343	0.007	0.394(0.199–0.765)
GS	-2.532e-02	3.335e-01	0.939	0.975(0.506–1.877)

*Abbreviations*: *GPC3* glypican 3, *CK19* keratin 19, *AFP* alpha fetoprotein, *EGFR* epidermal growth factor receptor, *RRM1* ribonucleotide reductase catalytic subunit M1, *BRCA1* breast cancer gene 1, *VEGF* vascular endothelial growth factor, *Ts* thymidylate synthase, *ARG* arginase, *GS* glutamine synthetase

Univariate analysis showed that the variables with *p* < 0.1 were tumor size, Ts,GPC3, p53, RRM1, BRCA1, and ARG. The results of multivariate logistic regression analysis revealed that tumor size (OR = 47.101, *p* < 0.001), GPC3 (OR = 14.256, *p* < 0.001), p53 (OR = 3.829, *p* = 0.018), RRM1 (OR = 3.134, *p* = 0.047), BRCA1 (OR = 4.314, *p* = 0.021), and ARG (OR = 0. 326, *p* = 0.044) were independent risk factors for MVI (Table 2).

**Table 2:** Multivariate logistic regression analysis of MVI occurrence in the training cohort

Variable	B	S.E	*P*	OR (95%CI)
Tumor size	3.852	0.668	< 0.001	47.101(14.391–205.023)
GPC3	2.657	0.643	< 0.001	14.256(4.458–57.281)
P53	1.342	0.567	0.018	3.829(1.308–12.385)
RRM1	1.143	0.576	0.047	3.134(1.034–10.124)
BRCA1	1.462	0.635	0.021	4.314(1.300–16.118)
ARG	-1.121	0.556	0.044	0.326(0.103–0.941)
Constant	-4.613	0.919	< 0.001	-

*Abbreviations*: *GPC3* glypican 3, *RRM1* ribonucleotide reductase catalytic subunit M1, *BRCA1* breast cancer gene 1, *ARG* arginase

The expressions of GPC3, p53, RRM1, BRCA1, and ARG protein in the MVI ( +) and MVI (-) groups were analyzed by immunohistochemistry. The expressions of GPC3, p53, RRM1, and BRCA1 in the MVI ( +) group were significantly up-regulated, while ARG expression was significantly down-regulated (Fig. S2).

### Construction and validation of the nomogram model

**Fig. 2:**
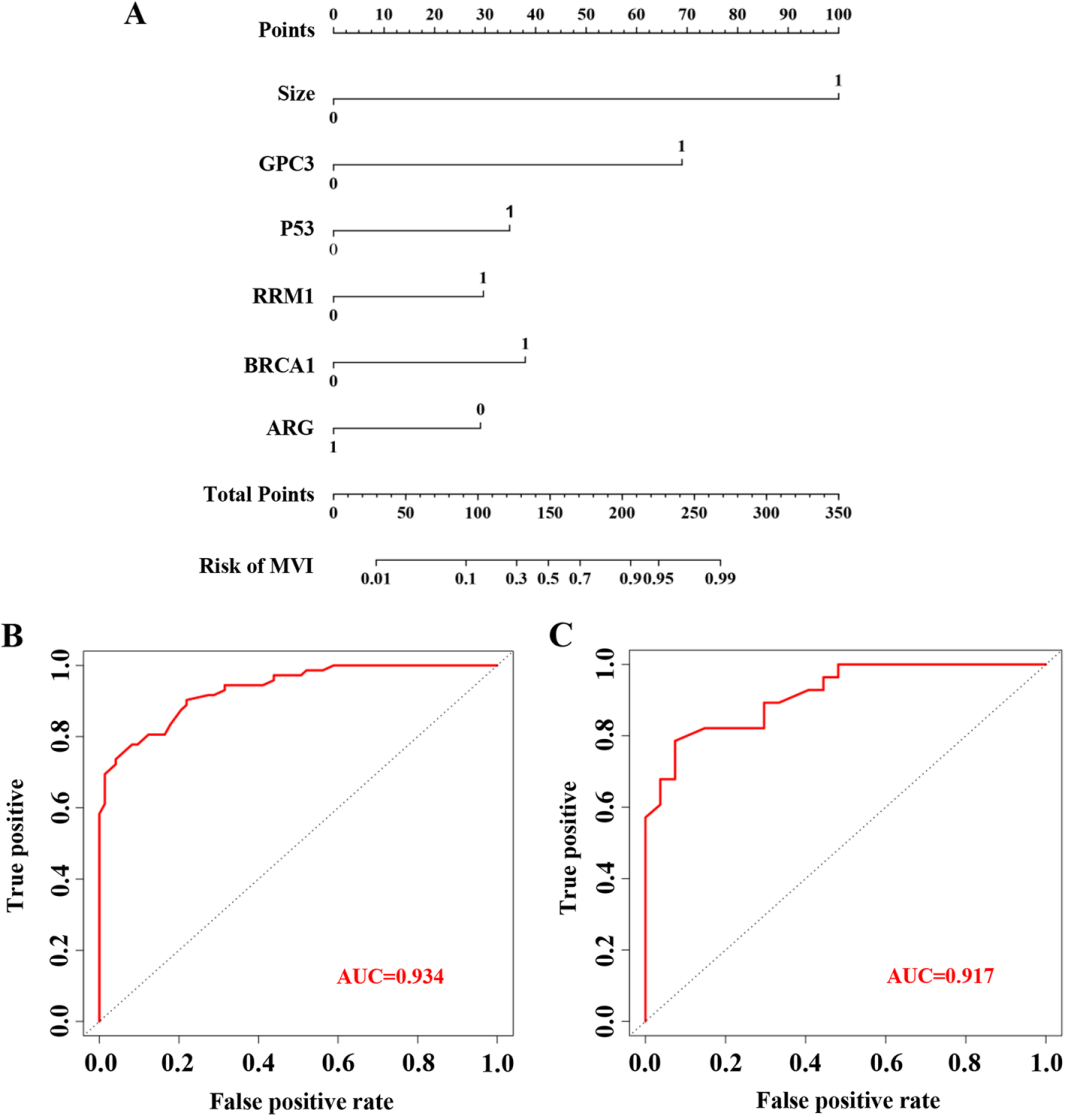
Construction of the nomogram model. A nomogram model to evaluate the risk of MVI occurrence in HCC (**A**). To use the nomogram, determine the position of each variable on the corresponding axis, draw a line to the points axis for the number of points, add the points from all the variables, and draw a line from the total points axis to determine the MVI probabilities at the lower line of the nomogram. ROC and AUC curves in the training cohort (**B**) and the validation cohort (**C**). Abbreviations: glypican 3 (GPC3); ribonucleotide reductase catalytic subunit M1 (RRM1); breast cancer gene 1 (BRCA1); arginase (ARG)

A nomogram model for MVI risk assessment was constructed (Fig. 2A) on the basis of the results of multivariate analysis. Tumor size had the greatest impact on MVI, with a maximum score of 100 points. The scores of GPC3, P53, RRM1, BRCA1, and ARG were 68, 34, 29, 35, and 25, respectively. Each patient was scored based on the risk factors, and the advantages and disadvantages of the nomogram model were evaluated by ROC analysis. Through this analysis, we found that the optimal cut-off value of 0.671 served as the prediction threshold for the risk scoring system. In other words, a total risk score of > 0.671 indicated MVI presence and a total risk score of ≤ 0.671 indicated MVI absence. ROC analysis revealed that the sensitivity of the risk prediction scoring system was 0.810, the specificity was 0.850, and AUC value was 0.934 (Fig. 2B). The AUC value in the validation cohort was 0.917, which was close to that in the training cohort (0.934) (Fig. 2C).

**Fig. 3:**
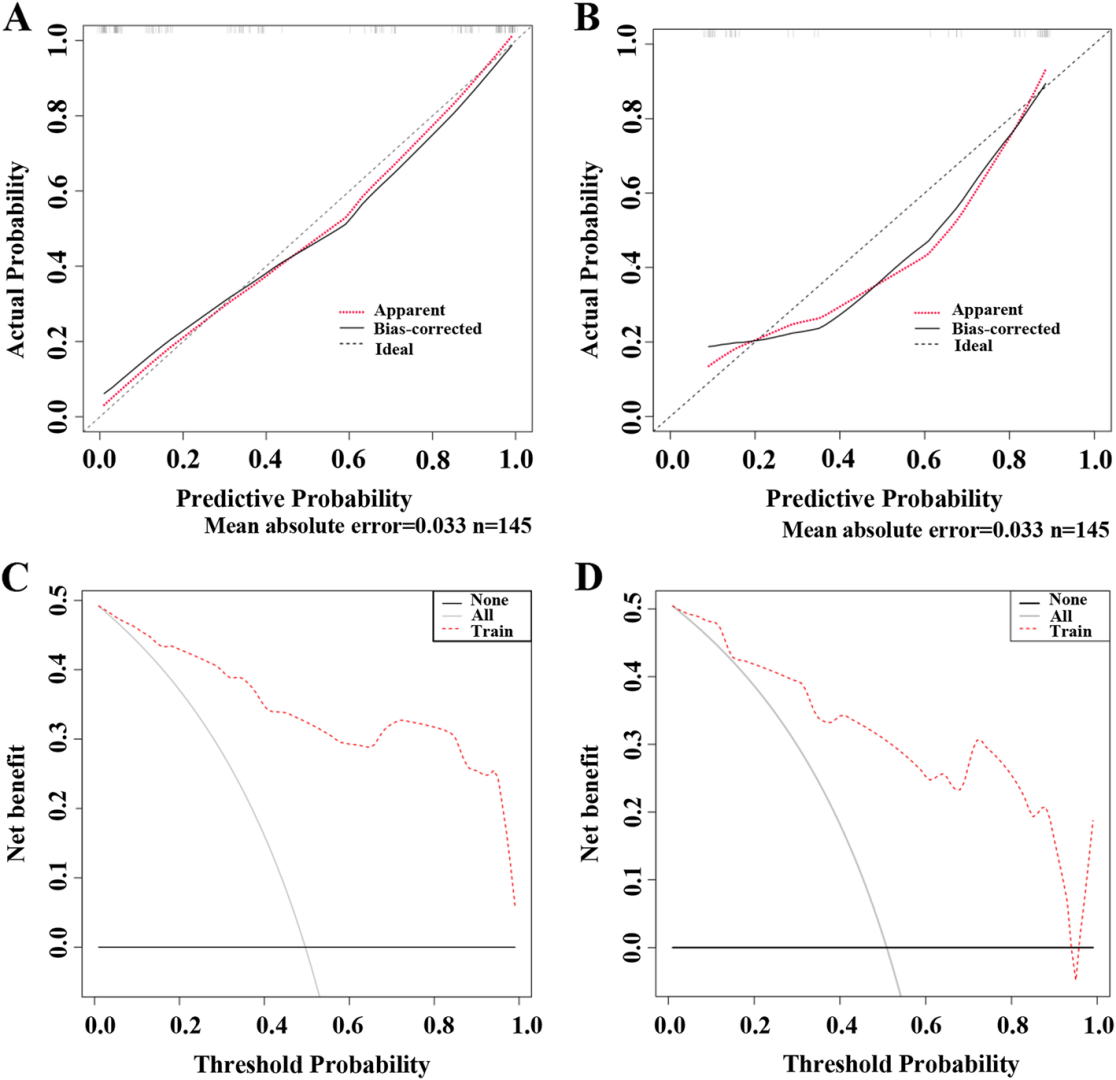
Validation of the nomogram model. The calibration curve of the logistic regression predictive model in the training cohort (**A**) and the validation cohort (**B**). The vertical axis represents the actual probability of MVI, and the horizontal axis represents the predictive probability of MVI. DCA analysis for the nomogram model in the training cohort (**C**) and the validation cohort (**D**)

The calibration curve was used to assess the accuracy of the nomogram model. The average deviation was discerned to be 0.033 (mean absolute error = 0.033, mean squared error = 0.001), and the predictive probability could better fit the actual probability. There was no significant difference in the Hosmer–Lemeshow goodness-of-fit test (χ2 = 10.367, *p* = 0.240) (Fig. 3A). The results of the training cohort suggested that discrimination and calibration were both high and met the requirements. In the validation cohort, the average deviation in accuracy was 0.052 (absolute error = 0.052, mean squared error = 0.004), and the predictive probability could also better fit the actual probability (Fig. 3B). DCA revealed that the curve of the nomogram model was far from the extreme curve and the benefit was higher than that of the extreme curve in the training and validation cohorts, indicating that the predictive model was effective (Fig. 3C and 3D).

## Discussion

With the rapid advances in radiology, more and more liver malignancies are being detected early and operated in a timely manner, which greatly improves patient survival. However, the recurrence rate of HCC remains high, and the 5-year survival rate of HCC patients is just 15% [14]. MVI is considered to be a main risk factor for the recurrence of HCC, and is closely related to poor overall survival [15]. So far, imaging studies, serological markers, and gene expression differences have been used to determine the occurrence of MVI. Pathological examination is the reference standard for MVI diagnosis, but this “gold standard” is not always reliable in the clinical practice. The diversity of HCC tumor specimens (such as less amount of tumor biopsy specimens, large number of surgically resected tumors and/or large tumor size), limited scope of routine glass slides, and the different diagnostic levels of pathologists may lead to MVI missed diagnosis. To improve the accuracy of MVI detection, this study applied logistic regression analysis in bioinformatics to establish a nomogram model based on tumor size and the differences in the expressions of five proteins in the HCC tissues. This measure was intended to promote the prediction of MVI and guide the clinical treatment decision making for HCC patients.

At present, the factors that predict MVI include the size and number of tumors, serum AFP, and the degree of lymphocyte infiltration in the liver tissue [16]. When compared with the single variable model, the multivariable model has better prediction ability for the occurrence of MVI. In this study, the clinical and pathological features closely related to the prognosis of HCC and the expression levels of 13 proteins, including GPC3, CK19, and vimentin, in the tumor tissue were selected for multivariate analysis. The results showed that tumor size, GPC3, P53, RRM1, BRCA1, and ARG were the independent risk factors for MVI. Previous studies have demonstrated that tumor size is closely related to the incidence of MVI and that the incidence increases with the increase in tumor diameter. Tumor size is an independent risk factor for MVI in HCC [17]. GPC3 is an independent risk factor for overall survival rate in patients with cancers and he incidence of MVI is higher in HCC patients with positive GPC3 expression [18]. High expression of P53 is related to the poor prognosis of triple negative breast cancer and ovarian cancer [19]. High expression of RRM1 can lead to poor prognosis in patients with non-small cell lung cancer [20]. RRM1 gene mutation leads to unrestricted proliferation of the tumor cells, accelerates angiogenesis, and promotes metastasis [21]. This study revealed that the expression of RRM1 is obviously increased in HCC with MVI ( +) tissues, suggesting that RRM1 could promote metastasis and affect the prognosis of the patients. Low expression of BRCA1 signifies worse prognosis in patients with triple negative breast cancer, while its high expression effectively inhibits invasion and metastasis [22]. Overexpression of ARG indicates poor prognosis in HCC, and is associated with the migration and invasion of cancer cells [23]. The above studies demonstrate that the six independent risk factors are closely related to the occurrence of MVI and have an impact on tumorigenesis and disease progression in the patients. Therefore, the inclusion of these risk factors in the construction of the MVI prediction model could help improve the pathological accuracy of MVI diagnosis in HCC patients.

Recently, artificial intelligence technology represented by machine learning has witnessed rapid progress and has been extensively applied in auxiliary diagnosis, prognostic analysis, and personalized diagnosis and treatment of diseases [24]. Common machine learning algorithms include logistic regression, support vector machines, random forest, artificial neural network, and light gradient boosting machine. Importantly, logistic regression has been extensively applied to predict the prognosis and disease recurrence in cancer patients [25–28]. Cucchetti et al. constructed an artificial neural network model on the basis of logistic regression analysis by taking into account the three factors of tumor number, diameter, and serum AFP level to accurately predict MVI in HCC patients [29]. However, this model requires specific software and equipment, which limits its application. In addition, logistic regression nomogram prediction model based on high AFP level, unsmooth tumor edge, enhancement around aneurysms, and imaging features of hepatobiliary phase (HBP) T1 weighted image and HBP-T1 map could be effectively used to evaluate the clinical results of MVI in HCC patients [30]. However, this method needs professional instruments, besides being expensive and having poor stability. In this study, the model for MVI occurrence was constructed based on the differences in tumor size, tumor differentiation, and differential analysis of protein expressions in the HCC tissues by logistic regression analysis. In the training cohort, ROC analysis demonstrated that the sensitivity, specificity, and AUC value of the model were 0.810, 0.850, and 0.934, respectively. In the validation cohort, the AUC value was 0.917, which was close to that of the training cohort. The above results implied that the model could efficiently predict the occurrence of MVI. Moreover, calibration curve and decision curve confirmed that the prediction model had good reliability and clinical application potential. In the follow-up study, we plan to further evaluate the accuracy and clinical value of the model in predicting MVI through multicenter validation.

This study had several limitations. First, the limited number of samples leaves the question open on the statistical power; therefore, we should continue to collect more samples. Second, this is a small-sample exploratory study in a single center. it is necessary to validate the results from other centers. A multicenter study with a larger population would contribute to verifying the present results of OSS in the future. Third, considering the sample was mostly composed by patients stricken by viral hepatitis, with few cases of metabolic diseases in this study. Therefore, using this model to predict MVI caused by dissimilar hepatic diseases may require further verification.

This study is the first to explore the nomogram prediction model based on differential protein expression in HCC tissues. This model has high reliability and clinical application potential and could therefore be used as an important method in the diagnosis and prediction of MVI.

## Conclusions

A nomogram model based on the clinical characteristics of HCC tumors and differential protein expression patterns was constructed in this study to predict MVI. This MVI prediction model can be used as an auxiliary diagnosis method to improve the accuracy of MVI diagnosis.

## Supplementary Information

**Additional file 1:** **Table S1.** Clinical characteristics of all patients.

**Additional file 2:** **Figure S1.** Background disease of HCC patients.

**Additional file 3:** **Figure S2.** Immunohistochemical staining analysis of GPC3, P53, RRM1, BRCA1, and ARG protein expression in MVI (-) and MVI (+) patients in the training cohort, 10×magnification. Abbreviations: glypican 3 (GPC3); ribonucleotide reductase catalytic subunit M1 (RRM1); breast cancer gene 1 (BRCA1); arginase (ARG).

## Data Availability

The datasets generated and analyzed during the current study are not publicly available due to health privacy concerns, but are available from the corresponding author on reasonable request.

## Abbreviations

HCC: Hepatocellular carcinoma
MVI: Microvascular invasion
ENO1: Enolase 1
HSP70: Heat shock protein 70
GPC3: Glypican 3
CK19: Keratin 19
AFP: Alpha fetoprotein
EGFR: Epidermal growth factor receptor
RRM1: Ribonucleotide reductase catalytic subunit M1
BRCA1: Breast cancer gene 1
VEGF: Vascular endothelial growth factor
Ts: Thymidylate synthase
ARG: Arginase
GS: Glutamine synthetase
