# Supplementary material for: A novel predictive model of microvascular invasion in hepatocellular carcinoma based on differential protein expression
Source: BMC Gastroenterol. 2023 Mar 27;23:89. doi: 10.1186/s12876-023-02729-z (PMC10041792; doi:10.1186/s12876-023-02729-z)
Supplement: Supplementary file 1 — Additional file 1: Table S1. Clinical characteristics of all patients. [file 12876_2023_2729_MOESM1_ESM.docx]

Table S1 Clinical characteristics of all patients.

| Variable | Expression | Training cohort（145） | Validation cohort（55） | P |  |
| --- | --- | --- | --- | --- | --- |
| MVI | 0 | 73(50.34%) | 27(49.09%) | 0.972 |  |
|  | 1 | 72(49.66%) | 28(50.91%) |  |  |
| Age |  | 53.880±9.720 | 53.090±10.490 | 0.615 | |
| Gender | Male | 107(73.79%) | 40(72.73%) | 0.992 | |
|  | Female | 38(26.21%) | 15(27.27%) |  |  |
| Tumor size | <5 cm | 76(52.41%) | 27(49.09%) | 0.743 | |
|  | >5 cm | 69(47.59%) | 28(50.91%) |  |  |
| Tumor  Differentiation | Poor | 23(15.86%) | 12(21.82%) | 0.116 | |
|  | Moderate | 111(76.55%) | 35(63.64%) |  |  |
|  | Good | 11(7.59%) | 8(14.54%) |  |  |
| GPC3 | 0 | 78(53.79%) | 27(49.09%) | 0.992 | |
|  | 1 | 67(46.21%) | 28(50.91%) |  |  |
| CK19 | 0 | 107(73.79%) | 40(72.73%) | 0.992 | |
|  | 1 | 38(26.21%) | 15(27.27%) |  |  |
| vimentin | 0 | 67(46.21%) | 25(45.45%) | 1.000 | |
|  | 1 | 78(53.79%) | 30(54.55%) |  |  |
| P53 | 0 | 83(57.24%) | 24(43.64%) | 0.075 | |
|  | 1 | 62(42.76%) | 31(56.36%) |  |  |
| AFP | 0 | 79(54.48%) | 33(60.00%) | 0.518 | |
|  | 1 | 66(45.52%) | 22(40.00%) |  |  |
| EGFR | 0 | 61(42.07%) | 23(41.82%) | 1.000 | |
|  | 1 | 84(57.93%) | 32(58.18%) |  |  |
| RRM1 | 0 | 61(42.07%) | 22(40.00%) | 0.878 | |
|  | 1 | 84(57.93%) | 33(60.00%) |  |  |
| BRCA1 | 0 | 48(33.10%) | 19(34.55%) | 0.946 | |
|  | 1 | 97(66.90%) | 36(65.45%) |  |  |
| VEGF | 0 | 65(44.83%) | 21(38.18%) | 0.418 | |
|  | 1 | 80(55.17%) | 34(61.82%) |  |  |
| Ts | 0 | 66(45.52%) | 23(41.82%) | 0.148 | |
|  | 1 | 79(54.48%) | 32(58.18%) |  |  |
| Ki 67 | 0 | 43(29.66%) | 11(20.00%) | 0.156 | |
|  | 1 | 102(70.34%) | 44(80.00%) |  |  |
| ARG | 0 | 16(11.03%) | 7(12.73%) | 0.878 | |
|  | 1 | 129(88.97%) | 48(82.27%) |  |  |
| GS | 0 | 66(45.52%) | 18(32.73%) | 0.088 | |
|  | 1 | 79(54.48%) | 37(67.27%) |  |  |

Abbreviations: glypican 3 (GPC3); keratin 19 (CK19); alpha fetoprotein (AFP); epidermal growth factor receptor (EGFR); ribonucleotide reductase catalytic subunit M1 (RRM1); breast cancer gene 1 (BRCA1); vascular endothelial growth factor (VEGF); thymidylate synthase (Ts); arginase (ARG); glutamine synthetase (GS).
