# Supplementary figures and images for: A novel predictive model of microvascular invasion in hepatocellular carcinoma based on differential protein expression
Source: BMC Gastroenterol. 2023 Mar 27;23:89. doi: 10.1186/s12876-023-02729-z (PMC10041792; doi:10.1186/s12876-023-02729-z)

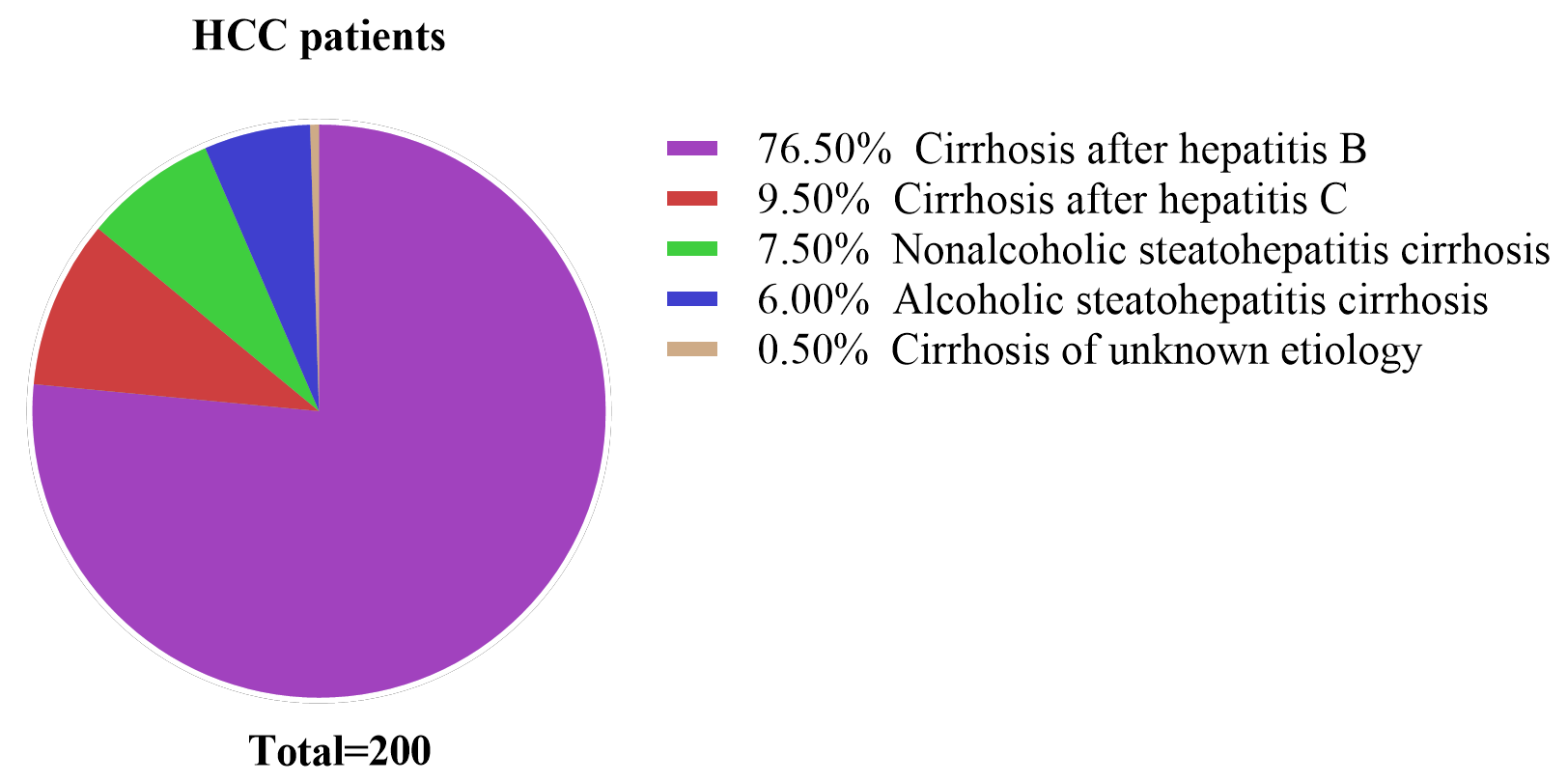


Figure S1 Background disease of HCC patients

Supplement: Supplementary file 2 — Additional file 2: Figure S1. Background disease of HCC patients. [file 12876_2023_2729_MOESM2_ESM.docx]
