# Supplementary material for: A novel predictive model of microvascular invasion in hepatocellular carcinoma based on differential protein expression
Source: BMC Gastroenterol. 2023 Mar 27;23:89. doi: 10.1186/s12876-023-02729-z (PMC10041792; doi:10.1186/s12876-023-02729-z)
Supplement: Supplementary file 3 — Additional file 3: Figure S2. Immunohistochemical staining analysis of GPC3, P53, RRM1, BRCA1, and ARG protein expression in MVI (-) and MVI (+) patients in the training cohort, 10×magnification. Abbreviations: glypican 3 (GPC3); ribonucleotide reductase catalytic subunit M1 (RRM1); breast cancer gene 1 (BRCA1); arginase (ARG). [file 12876_2023_2729_MOESM3_ESM.docx]

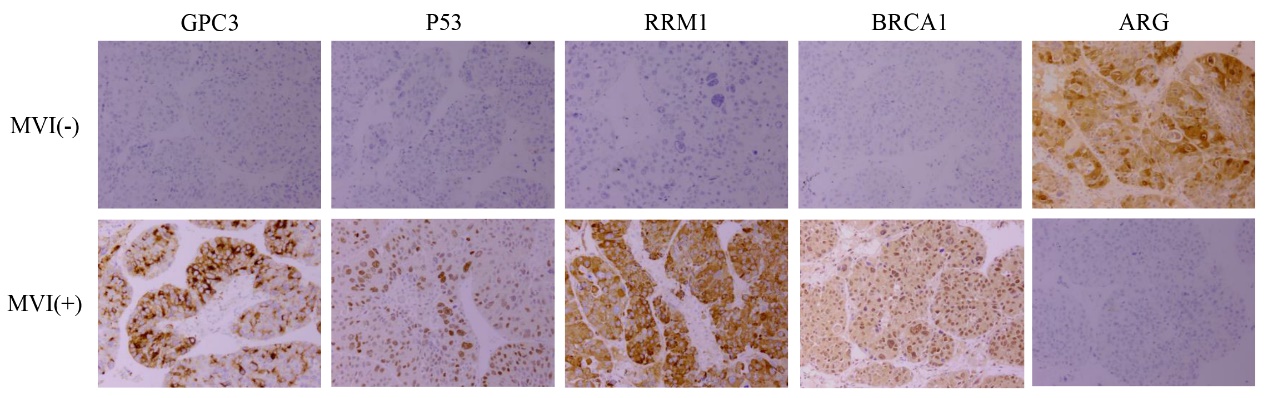


Figure S2 Immunohistochemical staining analysis of GPC3, P53, RRM1, BRCA1, and ARG protein expression in MVI (-) and MVI (+) patients in the training cohort, 10×magniﬁcation. Abbreviations: glypican 3 (GPC3); ribonucleotide reductase catalytic subunit M1 (RRM1); breast cancer gene 1 (BRCA1); arginase (ARG).
